# A Rare Case of Fahr's Syndrome With Bilateral Vocal Cord Paresis

**DOI:** 10.7759/cureus.28105

**Published:** 2022-08-17

**Authors:** Gokhan Demir, Gi Eun Kim, Abrar Yaser Alsayed, Saad Sameer, Madeha Khalid

**Affiliations:** 1 Internal Medicine, Hamad General Hospital, Doha, QAT; 2 Family Medicine, Hamad General Hospital, Doha, QAT

**Keywords:** fahr’s syndrome, fahr’s disease, normocalcemic hyperparathyroidism, neuropsychiatric symptoms, basal ganglia calcification, bilateral vocal cord paralysis, fahr’s disease or fahr’s syndrome

## Abstract

We will be discussing a very rare neurodegenerative disorder called Fahr’s disease, which is characterized by calcifications in the basal ganglia and other brain regions. Our case describes a 54-year-old lady presenting with abnormal aggressive behavior. Interestingly, in our case, the patient later developed vocal cord paresis, of which only one similar case has been reported before. CT was done, which showed typical extensive diffuse calcifications of the basal ganglia and other brain regions. Her labs were significant for normocalcemic hyperparathyroidism. During her stay in the hospital, she developed vocal cord paresis. In this case, she was managed in a multidisciplinary approach by medicine, neurology, psychiatry, and ENT. She improved significantly and was eventually discharged from the hospital. The rarity of Fahr’s disease and the atypical involvement of vocal cords made the management challenging; however, the multidisciplinary approach aided in achieving well-rounded patient care and clinical improvement.

## Introduction

Fahr’s disease is characterized by calcification of the basal ganglia and other gray matter structures, including cerebellar nuclei and punctate calcifications in the thalamus and cortex [1]. It usually starts in the fourth to fifth decade of life, with neurological and psychiatric manifestations. The disease’s etiology is currently unknown, although one study has proposed that mutations in the *SLC20A2* and *PDGFRB* genes are associated with Fahr’s disease [2,3]. When the basal ganglia calcifications are secondary to a known cause, the disease is referred to as Fahr’s syndrome.

The syndrome’s clinical manifestations feature mainly neurological and psychiatric findings. Vocal cord paresis is not of these, with medical literature reporting only one reported case of bilateral vocal cord paralysis associated with Fahr’s disease [4]. We report about the first known case of Fahr’s syndrome featuring vocal cord paresis.

The preferred imaging method to assess the extent of brain calcification is CT head scans, although head MRI can be used as well [5]. There is currently no therapy to reduce the progression of Fahr’s disease; therefore, symptomatic treatments are used, such as anti-depressants, anti-psychotics, and anti-Parkinson's drugs [5], as was done for our patient. In the case of Fahr’s syndrome due to calcium metabolism disorders, correction of calcium and phosphate levels can resolve symptoms such as mental retardation and seizures [5]. However, our patient had normal calcium, phosphate, and vitamin D levels. Other potential therapies that are still under investigation include bisphosphonates and vitamin D [6].

## Case presentation

A 54-year-old female was admitted to the hospital for abnormal behavior, which had started two weeks prior. She was combative and restless, as well as refusing to answer questions. This was the patient’s first time presenting with this behavior, although she had a 30-year history of severe depression and had been treated with multiple medications (paroxetine, mirtazapine, and quetiapine). Upon admission, she was non-compliant with home medications.

The patient was in her normal state but began to exhibit signs of a depressive episode one day before her abnormal behavior began. Two weeks prior to hospital admission, she developed bizarre, aggressive behavior that included harming herself and her family by taking her clothes off and screaming. Her family members reported that she had decreased oral intake, mainly spitting food and water when offered, and refused to take her medications. Her daughter denied any fever, headache, photophobia, phonophobia, fits, or tremors. The patient had no history of alcohol or recreational drug use. She did have positive asymptomatic coronavirus disease 2019 (COVID-19) results one month prior to the onset of her behavioral symptoms.

The patient’s past medical history was significant for multiple admissions to psychiatric hospitals for severe depression. She had been assessed at one such hospital 11 days prior to admission at our hospital and was recommended admission, but the family refused at that time. There was a significant family history of depression in the patient’s father and paternal uncle, but no family history of similar behavioral changes. She was also known to be hypertensive and was receiving amlodipine.

Upon assessing the patient, she was found to be disoriented to time, place, and person. Her speech was incoherent, and she did not follow commands and was otherwise uncooperative. A neurological assessment did not reveal any significant findings.

Upon physical examination, the patient was alert and agitated but did not respond to questions. Ecchymosis was significantly bilaterally present on her upper and lower limbs. The patient did not have any signs of pseudohypoparathyroidism such as short 4th metacarpals and short limbs. Her vital signs were within normal limits.

Laboratory investigations revealed a white blood cell count of 11.4 K/μL, hemoglobin of 10.7 g/dL, and mean corpuscular volume (MCV) of 72.3 fL. A peripheral smear showed mild hypochromic microcytic anemia with slight anisocytosis. Few RBC fragments and ovalocytes with mild thrombocytosis were seen. The patient’s coagulation profile was unremarkable. Other significant investigations showed normal low adjusted calcium at 2.20 mmol/L, parathyroid hormone (PTH) of 117 pg/mL, and vitamin D of 25 ng/mL, which show a picture of normocalcemic hyperparathyroidism (Table 1). Unfortunately, PTH and vitamin D were not repeated during the hospital course. Autoimmune antibodies (antineutrophil cytoplasmic antibody (ANCA), antinuclear antibody (ANA), autoimmune antibody panel, and anti-proteinase 3ab) were all negative. Complement proteins C3 and C4 were within normal limits, and a COVID-19 rapid test was negative.

**Table 1:** Summary of the results of laboratory findings Adjusted serum calcium (mmol/L) = (0.02 * (40 - patient's albumin) + serum calcium.

Laboratory investigation	Unit	On admission	On discharge	Reference range
White blood cell count	K/μL	11.7	13	4.0-10.0
Hemoglobin	gm/dL	10.7	12.5	12.0-15.0
Mean corpuscular volume	fl	72.3	75.5	83.0-101.0
Prothrombin time	seconds	10.6	10.3	9.7-11.8
Partial thromboplastin time	seconds	25.1	25.3	24.6-31.2
International normalized ratio		1	1	0.8-1.2
C-reactive protein	mg/L	17.1	8.2	0.0-5.0
Alanine transferase	U/L	44	43	0-33
Aspartate transferase	U/L	31	28	0-32
Alkaline phosphatase	U/L	91	112	35-104
Albumin	gm/L	38	29	35-50
Urea	mmol/L	4.6	6.2	2.5-7.8
Creatinine	umol/L	60	66	44-80
Sodium	mmol/L	146	138	133-146
Potassium	mmol/L	3.3	4.1	3.5-4.3
Chloride	mmol/L	107	102	95-108
Vitamin D	ng/mL	25		
Parathyroid hormone, intact	pg/mL	117		15-65
Serum calcium	mmol/L	2.37	2.32	
Adjusted serum calcium	mmol/L	2.41	2.54	2.20-2.60
Magnesium	mmol/L	0.82	0.81	0.70-1.00
Phosphorus	mmol/L	1.11	1.29	0.8-1.50

**Figure 1:**
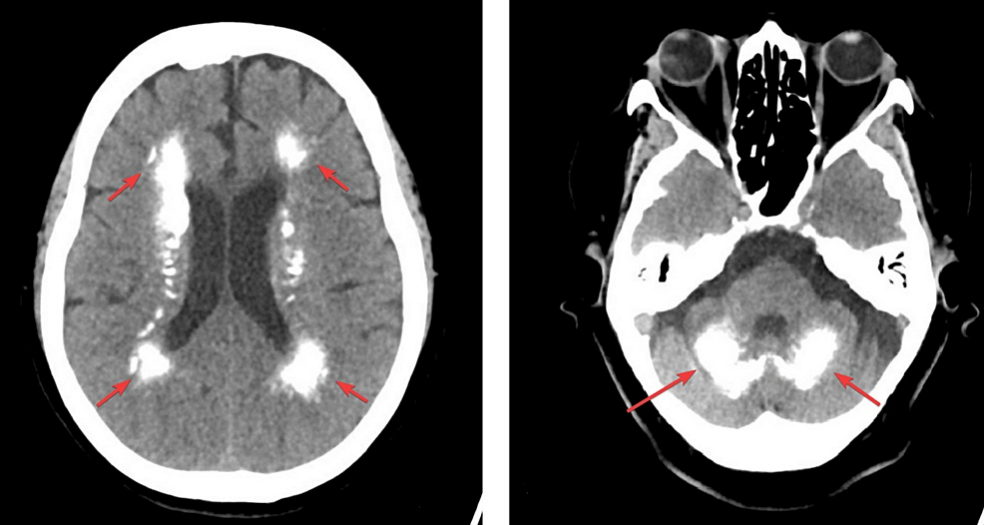
CT scans of the head without contrast showing extensive diffuse calcification involving the basal ganglia, centrum semiovale, posterior occipital lobes, and the cerebellum. The occipital lobes’ calcification involves the cortex.

CT of the head showed basal ganglia calcifications, with additional scattered calcifications in the periventricular and occipital lobes (Figure 1).

**Figure 2:**
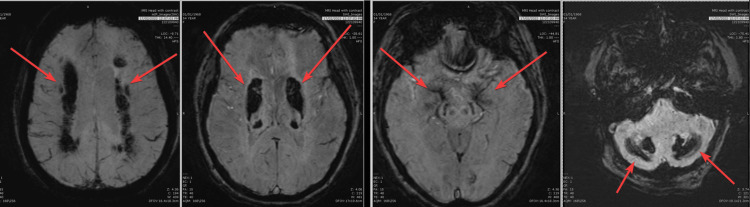
MRI susceptibility weighted imaging sequence demonstrates severe and intense calcification along the bilateral cerebellum, brain stem substantia nigra, thalamus, basal ganglia periventricular white matter, centrum semiovale, and occipital cortical gray matter.

A brain MRI was later conducted and showed bilateral symmetrical extensive coarse calcification involving the bilateral basal ganglia, thalami, corona radiata, centrum semiovale, bilateral occipital parasagittal region, occipital lobes, and the cerebellum (Figure 2). All of these features are known for Fahr’s disease.

**Figure 3:**
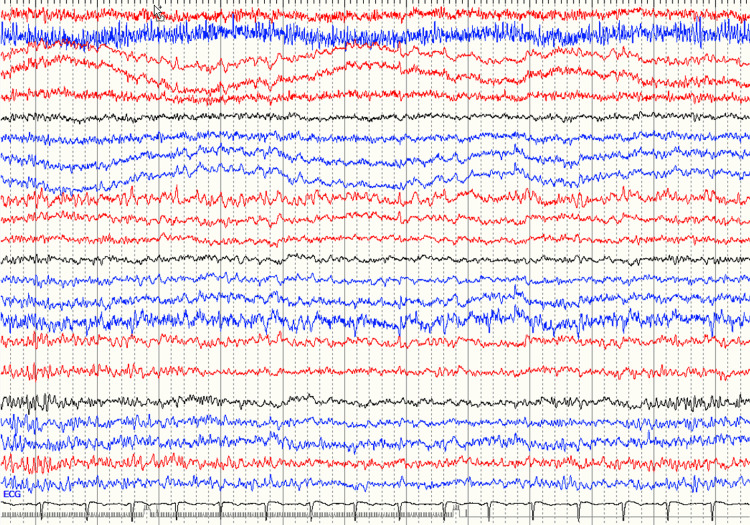
During brief maximal alertness, the dominant background rhythms consist of moderate amounts of bilaterally symmetrical 30-50 microvolts irregular 9-10 Hz alpha activity seen over the posterior head region(s). It does intermix at times with slower waves in the high theta frequency range. All these findings are suggestive of a mild non-specific diffuse disturbance of cerebral cortical activity.

An electroencephalogram (EEG) study was done multiple times on the patient and was continuously suggestive of a mild, nonspecific diffuse disturbance of cerebral cortical activity. No epileptiform discharges were seen (Figure 3).

Further investigations done over the next few days included cerebrospinal fluid (CSF) for cell count, mycobacteriology, acid-fast bacillus smear virology panel, and fungal culture, which were all negative. The patient’s CSF glucose level was 3.91 mmol/L, and her CSF protein, albumin, CSF oligoclonal banding, IgG, and IgG index levels were all insignificant. An additional encephalopathy autoimmune evaluation CSF sent to Mayo Clinic Laboratories was insignificant.

The patient was put on a nasogastric tube, as she was spitting and not swallowing any of the food provided to her. She mostly remained in bed not moving, with an unstable gait that developed if she was moved. She was continuously agitated, disoriented, and aggressive. The psychiatry team started her on haloperidol 2 mg twice a day (bid) pro re nata (PRN), paroxetine 30 mg, mirtazapine 15 mg, and quetiapine 300 mg at bedtime.

Two months after the hospital admission, the patient developed noisy breathing (biphasic stridor), productive cough, and difficulty breathing, and her oxygen saturation dropped, requiring a 5 L face mask. A fiberoptic exam done by the otolaryngology team showed that the patient had unilateral right vocal cord palsy with left vocal cord limited abduction in the paramedian position. Edema of the aryepiglottic fold in the arytenoid and interarytenoid area was observed. The patient was thus started on 8 mg dexamethasone intravenous (IV) two times a day (bid) and budesonide nebulization, after which she started showing improvement.

One week after developing stridor, the patient improved significantly. Her consciousness improved, she was less agitated, and the psychiatry team recommended keeping her on intramuscular olanzapine as needed for agitation and quetiapine. She was also reassessed by the otolaryngology team again and was recommended to continue a steroid inhaler and IV dexamethasone, as arytenoid edema was still present based on a follow-up fiberoptic exam.

The patient was discharged on quetiapine and sertraline and followed up with psychiatry. She also underwent a follow-up nasopharyngoscopy nearly one month after the stridor event. It showed bilateral vocal cord paralysis near the midline position with sluggish movement of the right vocal cord, a small open gape in the abduction position, and severe laryngopharyngeal reflux with arytenoid edema.

## Discussion

The initial differential diagnosis list for bilateral basal ganglia calcification included metabolic (hypoparathyroidism), autoimmune (encephalitis), and genetic diseases (Fahr's syndrome). We thought that infectious and autoimmune causes were unlikely for the following reasons such as unremarkable CSF cell counts, negative fungal and bacterial cultures from CSF, and negative autoimmune antibodies in CSF. EEG did not show any suggestive findings of encephalopathy. In addition, the patient did not respond to the trial of intravenous immunoglobulin and steroid course, which are the main treatments for autoimmune encephalitis. We also excluded the possibility of hypoparathyroidism by finding high PTH levels.

This case of Fahr’s syndrome is secondary to normocalcemic hyperparathyroidism. Although, our patient was on vitamin D supplements before admission, which could have altered the calcium values from low to normal levels. Normocalcemic hyperparathyroidism occurs when there are persistently elevated PTH and normal calcium levels (> three months) without any secondary causes of elevated PTH [7]. Common secondary causes of elevated PTH include medications, hypovitaminosis D, renal insufficiency, malabsorption, and low calcium intake [7]. In turn, Fahr’s syndrome is caused by calcium metabolism disorders [6]. Although the most common cause is hypoparathyroidism, hyperparathyroidism is another known cause of Fahr’s syndrome [8], as seen in our patient. Additionally, the patient had a history of psychiatric disorders, predominantly depression, but before the presentation of Fahr’s disease, she never had any episodes of erratic behavior. Whether the gray matter calcification from Fahr’s disease was the real cause behind her depression remains unknown.

A case report by Ghogare and Nemade describes an instance of Fahr’s syndrome presenting similarly to our case, with cognitive impairment and behavioral abnormalities but no motor symptoms [9]. In that case report, the patient’s main symptoms included not being able to recognize relatives, becoming dependent for daily activities, as well as irritability, crying episodes, and stubbornness. The patient presented relatively young at the age of 38, and their CT brain scan showed calcification only in the basal ganglia [9]. In contrast, our patient presented relatively late in life and had extensive calcifications. A review by Peters et al. states that patients who become symptomatic early in life present with cognitive impairment while patients who become symptomatic later in life exhibit mainly motor symptoms [6], which did not comply with our findings considering that psychiatric symptoms were the main complaint in our case.

Another interesting aspect of our case is the development of bilateral vocal cord paresis, which is a very rare manifestation of Fahr’s syndrome. In our case, the initial assessment of the otolaryngology team was bilateral vocal cord paralysis, but we were of the opinion that it was very unlikely for someone who has bilateral vocal cord paralysis to have been improved with only oxygen, steroids, and nebulizers provided that patient's family did not agree for tracheostomy. The prognosis of the situation is more suggestive of bilateral vocal cord paresis instead of paralysis.

Causes for bilateral vocal cord paresis include radiation therapy, prolonged intubation, surgical trauma to the vagus or recurrent laryngeal nerves, glottic and supraglottic malignancy, central nervous system pathologies such as stroke, CNS tumors, multiple sclerosis, and other conditions that may affect the nuclei of the vagus nerves and lead to paresis or paralysis of the vocal cords. In our patient, none of them were applicable except the possible effect of intracranial calcification caused by Fahr's syndrome on the nuclei of the vagus nerves.

There is only one similar case report in the literature on bilateral vocal cord paralysis presenting in Fahr’s syndrome. In contrast to our study, the other patient had undergone a total thyroidectomy 30 years prior. Our patient did not have any history of neck surgeries; however, she had undergone repeated insertion of a nasogastric tube due to her recurrent removal of the tube. Goutham et al.’s case also underwent tracheostomy as part of treatment for cord paralysis and improved [4], but our patient improved only after receiving IV dexamethasone and budesonide nebulization, as her family refused a tracheostomy, which suggests vocal cord paresis rather than paralysis.

## Conclusions

Fahr’s disease is a rare neurological condition characterized by calcification of the basal ganglia. It has a wide variety of clinical presentations depending on the specific areas in the basal ganglia affected by the calcification and its extent, though unfortunately, not enough research has been reported to effectively classify patients based on their clinical presentations. Currently, we can optimize treatment outcomes through a multidisciplinary approach involving neurologists, psychiatrists, and social workers.

Moving forward, we need to better understand if there is a genetic predisposition to Fahr’s disease and if we can detect it before it manifests as an irreversible syndrome. This will also help determine if it has any associations with other psychiatric or neurological conditions.
